# The climate impacts of healthcare digitalization: A scoping review

**DOI:** 10.1177/20552076251364666

**Published:** 2025-08-25

**Authors:** Ari Nissinen, Atte Pitkänen, Anton Barchuk, Aida Hosseinian, Annika Johansson, Matti Kaisti, Jaakko Karvonen, Pekka Marttinen, Hans Moen, Emilia Peltola, Laura Sokka, Kari A. O. Tikkinen

**Affiliations:** 1 Climate Solutions Unit, 73144 Finnish Environment Institute (Syke), Helsinki, Finland; 2 Faculty of Medicine, 60655 University of Helsinki, Helsinki, Finland; 3 Department of Medical Informatics, 6993 Erasmus Medical Center, Rotterdam, The Netherlands; 4 Climate Solutions Unit, Finnish Environment Institute (Syke), Oulu, Finland; 5 Department of Computing, 8058 University of Turku, Turku, Finland; 6 Circular Economy Solutions Unit, 73144 Finnish Environment Institute (Syke), Joensuu, Finland; 7 Department of Computer Science, Aalto University, Espoo, Finland; 8 Department of Mechanical and Materials Engineering, University of Turku, Turku, Finland; 9 Department of Urology, 3836 University of Helsinki and Helsinki University Hospital, Helsinki, Finland; 10 152642 Department of Health Research Methods, Evidence and Impact, McMaster University, Hamilton, ON, Canada; 11 Department of Surgery, 60667 Päijät Häme Central Hospital, Lahti, Finland

**Keywords:** Digitalization, digital health, healthcare, hospitals, telemedicine, diagnoses, medical appliances, artificial intelligence, carbon footprint, greenhouse gas emissions, leadership, procurement

## Abstract

**Objective:**

The rapid digitalization of healthcare has implications for its carbon footprint. The goal of this scoping review was to identify how digitalization is proceeding in healthcare and the mechanisms through which it can affect the climate impacts of healthcare.

**Methods:**

The scoping review was conducted following PRISMA guidelines and utilized the databases Web of Science and PubMed to identify literature on the climate impacts of digitalization in healthcare. The literature search identified 32 relevant studies. In addition, diagnostics, overdiagnosis, self-tracking technologies, and artificial intelligence (AI) were identified as key topics not sufficiently represented in the literature review, and related articles were added into the material.

**Results:**

Most carbon footprint analyses focused on telemedicine solutions, but research topics also included health informatics, education, diagnoses, overdiagnosis, treatments, medical appliances, robotics, and AI. Regarding telemedicine, the carbon footprint of the virtual consultations ranged between 0·005 and 3 kgCO2e, while the in-person healthcare clinic visits ranged between 0·57 and 178 kgCO2e depending on the mode of transport used, the difference in the carbon footprint being 79–99%. Although the transparency of carbon footprint analyses was often low, system boundaries were often different, and the evidence for digitalization decreasing or increasing climate impacts was limited.

**Conclusions:**

The findings highlight the need for future research on these topics and that leadership and setting strategic goals in particular were missing in the literature. Our scoping review also presents a framework for digitalization-related topics and means for advancing a lower carbon footprint in healthcare.

## Introduction

Digitalization is a megatrend and a great driving force for changes in all sectors of society.^1^ Digitalization is changing societies and economies via the use of digital technologies (i.e. the widespread deployment of digital information and communications technologies (ICTs), where *an ICT* is any means of storing, processing, or transmitting units of information).^2^ As the German Advisory Council on Global Change (WBGU)^1^ put it, “sustainability pioneers must seize the opportunities offered by digitalization and, at the same time, strive to minimize its negative climate impacts.”

Because healthcare is a crucial sector in society, providing vital services to people, it is important to develop low-carbon solutions for the sector. The share of gross domestic product spent on healthcare varies across countries, ranging from 4% in India to 17% in the United States (being 10–11% in the EU and Brazil). Procurement and investments cover energy, transport, the construction of buildings, and a wide spectrum of products and services.^3^

Consequently, the climate impact of healthcare is also relatively large, accounting for 4**·**4% of the global greenhouse gas (GHG) emissions.^3,4^ This varies by country, ranging from 1·5% to 7·6% of national carbon footprint.^3^ In the roadmaps following the Paris Agreement and leading the way to climate neutral societies, it is essential to focus on sectors with such a significant impact.

In terms of data storage and processing, health data have been estimated to account for 30% of the total data generated worldwide.^5^ The wider adoption of digital health technologies is currently considered inevitable, and standardized environmental impact assessment methods and tools have been proposed as the key to guiding decision makers and healthcare professionals toward the environmentally sustainable adoption of digital health.^6^ Developments in telehealth—or, more broadly, in virtual care—have been widely studied.^7–10^ Gray^11^ and Lokmic-Tomkins et al.^12,13^ reviewed a variety of potential changes in healthcare driven by digitalization.

Climate impact analyses are made on very different scales, ranging from the whole healthcare sector to single healthcare products. However, we noticed an absence of a comprehensive synthesis. This scoping review aims to identify and analyze how digitalization is proceeding in healthcare and the mechanisms through which it can affect healthcare's carbon footprint.

## Methods

The analysis is primarily based on a scoping review that follows the PRISMA guidelines^14^ (see Appendix A for the PRISMA Checklist)—designed to identify digitalization cases with impacts on the carbon footprints of healthcare—laying the ground for the topics presented in the framework in Chapter 4. In addition, diagnostics, overdiagnosis, self-tracking technologies, artificial intelligence (AI), and innovations were identified as key topics related to digitalization but insufficiently represented in the literature review. These fields are developing fast, and the authors selected articles that are relevant for the discussion although not identified in the search (e.g. due to missing search term digital*). Additionally, global and multi-national values for healthcare's carbon footprint^3,4^ and a thorough analysis of the sources of the carbon footprint of England's healthcare^15^ were examined.

### Search strategy and selection criteria

The databases Web of Science and PubMed were used to search for literature for this scoping review. Articles published before March 1, 2024, were included. The search terms were identified through relevant review and research articles and were refined along the search process. The initial search terms targeted digitalization and digital solutions with keywords related to healthcare, GHG emissions, and carbon footprint. During the screening process, telemedicine was identified as a key area where digitalization impacts on healthcare's climate footprint. Therefore, telemedicine and related terms, such as *virtual consulting*, were added into the database search. The search strategy is reported in full in Supplemental Table S1 (see Appendix B).

The database search yielded 474 articles. After removing duplicates, the titles and abstracts of the records were screened based on the following inclusion criteria:

1. The article presents an analysis of carbon footprints.
2. The article is related to digitalization in healthcare.
3. Regarding telemedicine, the article considers the carbon footprint of a digital health service and reports the carbon footprint results per consultation or visit.

A full text examination was conducted for articles that passed the initial screening. Only peer-reviewed English–language articles published in scientific journals were considered, and gray literature was excluded from the review.

**Figure 1.**
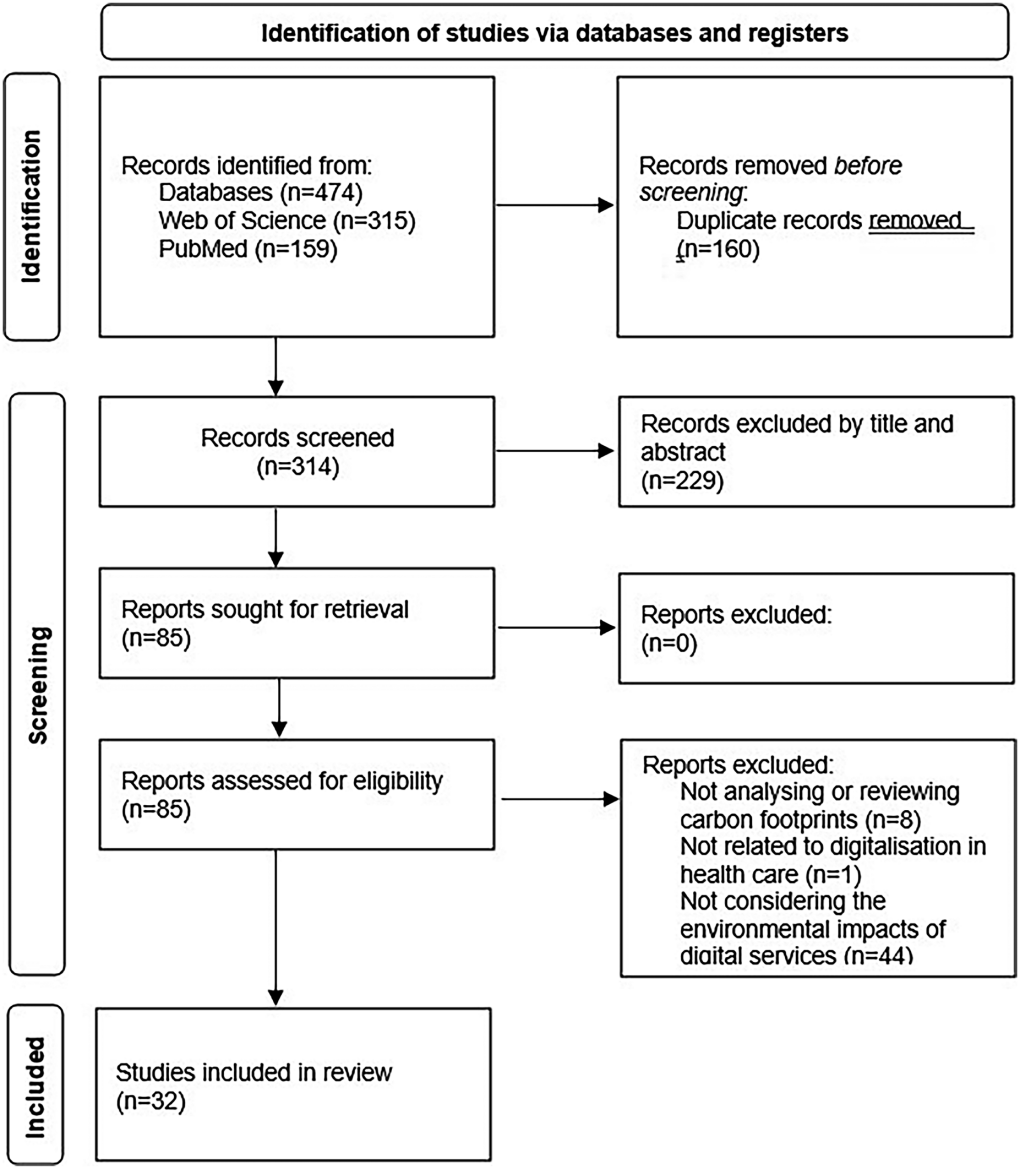
A PRISMA flow chart of record retrieval and selection.

Following the screening process, 16 original research articles were included in the review. Due to this low number of original research articles, 16 additional articles were included: 11 review articles and 5 special articles (commentaries and perspectives). This led to the 32 articles described in Supplemental Table S2 (see Appendix C). Each article was reviewed by two researchers who examined the analyzed topics, key findings, and carbon footprint results. The whole record retrieval and selection process is presented in Figure 1.

Data from each reviewed article was independently extracted to an Excel spreadsheet by two researchers. The data variables included the title, the authors, the year of publication, the study context, the digitalization topic considered, the type of analysis, the key findings, the carbon footprint impact of the digital solution, and the magnitude of the possible carbon footprint increase or decrease. From the original telemedicine studies, data were also sought on the system boundaries of the study and on the assessment methods of the emissions from travel and telemedicine solutions.

We compiled the carbon footprint results from these studies thematically in Supplemental Table S3 (see Appendix D). However, we only considered the carbon footprints as indicative of the order of magnitude rather than exact carbon footprint values. We used the term *low-carbon* instead of terms like *zero-carbon* and *net-zero*. See more in Table S3.

### The role of the funding source

The funding sources had no role in the study design; in the collection, analysis, and interpretation of data; in the writing of the paper; nor in the decision to submit the paper for publication.

## Results

### Literature review

#### Methodological shortcomings in the carbon footprint analyses of healthcare

The methodologies used in the carbon footprint studies of the healthcare sector have been strongly criticized. Lokmic-Tomkins et al.^12^ reviewed 13 studies, and no standardized methods or validated tools were identified to systematically determine the environmental sustainability of a digital health intervention over its full life cycle from conception to realization. Lange et al.^16^ developed a transparency checklist and found that the average transparency score for 23 studies was low, at just 38% (min. 14%, max. 68%), mostly due to the lack of reporting system boundaries, life cycle phases, allocation procedures, and the temporal representativeness and completeness of data. They concluded that the evidence for digitalization decreasing healthcare carbon footprint is so far weak due to this low transparency (see Table S3 in Appendix D for more details).

#### Review findings: original research articles

The majority of the reviewed original research articles (nine out of 17) studied the impact of telemedicine on the carbon footprint of healthcare. Only studies accounting for the GHG emissions of telemedicine services were included in the review (i.e. studies that only considered the GHG emission reductions related to avoided travel were excluded). Assessments of avoided travel are valuable but may exclude important sources of emissions related to telemedicine's provision. Furthermore, only studies that presented telemedicine-related GHG emissions per comparable functional unit (e.g. one consultation) were included.

The studies of telemedicine had methodological differences in the evaluation of the patient and staff travel. Five of the studies^9,10,26–28^ assumed that patients would travel by car to the healthcare facilities, and two of these studies^9,10^ had a threshold for a distance, after which patients would instead travel by plane. Two studies^7,29^ gathered data from a patient sample to confirm their traveled distance and mode of transport for appointments. Schmitz-Grosz et al.^30^ used a modal split describing the average share of transport modes over a traveled distance for their assessment of patient travel emissions, and Morcillo Serra et al.^31^ combined round-trip distance data from a private healthcare company and the average national modal split data. Only one study^26^ accounted for staff travel.

Additional differences emerged in how the telemedicine studies accounted for the emissions associated with providing virtual care, including the appliances and data connections needed and increased electricity consumption at home. Four studies^7,10,29,30^ only accounted for the electricity consumption of virtual appointments, which resulted in a very low-carbon footprint for telemedicine provision (< 0.04 kgCO_2_e per virtual appointment) and a greater than 98% carbon footprint reduction through telemedicine. The other five reviewed studies,^9,26–28,31^ which accounted more comprehensively for telemedicine equipment and data transfer, found a carbon footprint between 0**·**13 and 3 kgCO_2_e per virtual appointment, resulting in a footprint reduction of between 79% and 99% through telemedicine. Furthermore, two studies considered the carbon footprint associated with products such as protective equipment, paper, and hand sanitizers used during in-person meetings, which are not applicable to remote consultations.^9,26^

The positive effect of telemedicine on carbon footprints should also be analyzed in a broader context, focusing on which diseases can be effectively evaluated through this approach and pinpointing situations where it may result in delays in receiving appropriate treatment.^7,9^ Such delays could, in turn, contribute to a higher carbon footprint and compromise patient well-being. The optimization of staff travel in a hybrid telehealth service was demonstrated through mathematical modeling by Palmer et al.^32^

Other than telemedicine, only a few original research articles quantified changes in the carbon footprint by a digital intervention compared with baseline healthcare provision. These studies showed that 3D-printed pharmaceuticals can reach comparable levels of GHG emissions as those of traditional tableting through, for example, machine learning (ML).^33,34^ They also found that, currently in India, an electronic medical record system causes tenfold emissions compared with a paper record-keeping system but can reach on par emissions when renewable electricity is used.^35^ Additionally, autonomous AI can provide significant GHG emission reductions in healthcare through reduced travel and reduced facility needs.^36^ Virtual continuous medical education can provide a 99**·**7% emission reduction compared with an in-person event, mainly through avoided travel,^37^ and telepsychiatry can reduce emissions through avoided patient and provider travel.^38^

In other studies, a life cycle assessment was conducted to assess several environmental impact categories of the life cycle of a digital health communication service within an elderly living scheme, and the results showed that the electricity and printed circuit boards of the electronical equipment were found to contribute most to the carbon footprint.^39^ Additionally, Vafaei Sadr et al.^40^ identified strategies for reducing the carbon footprint associated with the use of deep learning in digital pathology.

#### Review findings: previous reviews of the climate impacts of healthcare digitalization

Review studies have shown sizeable GHG emission reductions through the travel avoided by using telemedicine (e.g. through virtual consultations) in healthcare.^8,16–19^ Rodler et al.^17^ reviewed the literature on the carbon footprint impacts of telemedicine and found a significant correlation between telemedicine and decreased GHG emissions. Most of the 48 reviewed studies only measured saved travel distances and converted them into GHG emissions, and only 9 studies considered the carbon footprint of the telemedicine services.

Ravindrane and Patel^18^ found 14 original research articles that reported emissions reductions through telemedicine. However, only three of the studies accounted for the telemedicine equipment. Rahimi-Ardabili et al.^20^ found that the literature mostly focused on telemedicine's potential to reduce patient and staff travel. They argued that while telemedicine has had the highest mitigation impact of digital health interventions to date, telemedicine studies suffer from inconsistency and a narrow scope. Lokmic-Tomkins et al.^12^ found that while the literature primarily focused on telehealth emissions, it also included topics such as electronic health records, e-prescriptions, and robotic surgery.

Samuel and Lucassen^21^ reviewed data-driven health research. They emphasized choosing the computing facility and any associated hardware carefully and increasing the efficiency of the code used to analyze the data, piloting any “energy-hungry” algorithms on smaller datasets first. They also noted that 100 megatons of GHGs are emitted from the digital sector each year, and the storage and processing of health-related data is the fastest-growing sector in the datasphere. In line with these results, Das and Chandra^22^ concluded that well-optimized AI training and execution can significantly decrease the climate footprint. They highlighted methods such as predictive maintenance, employing tiny AI models for data analysis with reduced energy requirements during computation and eliminating unnecessary consultations and subsequent facility needs. They additionally emphasized anomaly detection in the energy consumption of both specific devices and healthcare facilities overall.

Schmidt and Bohnet-Joschko^25^ reviewed research on hospitals’ carbon footprints and the possibilities to mitigate emissions and found that digital transformation is a key factor in implementing climate actions. They identified leadership as the first of 10 sustainability goals as it is essential to achieve a long-term organizational culture where environmental health becomes a strategic priority. However, this aspect was absent in the 21 studies they reviewed. They concluded that future research should focus on analyzing the role of leadership and management in mitigating GHG emissions. Furthermore, they noted that leadership in hospitals includes top–down strategic approaches and departmental and individual initiatives. The other nine sustainability goals that were present in the studies follow (the number of studies featuring each goal appears in brackets): energy (17), transportation (15), waste (14), purchasing (6), water (6), chemicals (4), pharmaceuticals (4), food (3), and buildings (2).

#### Review findings: special articles discussing healthcare digitalization

Lokmic-Tomkins et al.^13^ examined in a perspectives article a broad spectrum of digital health technologies, including medical devices and wearables, digital infrastructure, electronic medical records and prescribing, telehealth, mobile applications, augmented intelligence, and the Internet of Things (IoT). They offered redesign examples aimed at reducing the carbon footprint of these technologies and healthcare systems. They argued that energy- and resource-efficient cloud computing and the use of renewable energy are essential solutions for mitigating the emissions generated by digital health technologies. On the contrary, in a commentary article, Thompson^23^ highlighted that the harmful environmental impacts of digital healthcare have often been overlooked.

Similarly, Fragão-Marques and Ozben^24^ proposed in an opinion paper that, while telehealth has the potential to decrease healthcare's carbon footprint, more focus on the adequate planning of the large-scale implementation of digitalization is needed. For example, interactions between the actors that populate healthcare organizations can be enhanced, and resource sharing can be done more efficiently. Live maps can assist logistic companies and provide solutions for clinical laboratories to distribute and share reagents within a laboratory network. Additionally, health information centers can have updated information from both public and private hospitals concerning bed availability, material, equipment, and medicines.

Gray^11^ focused in a mini review on health informatics and concluded that a low-carbon health ICT infrastructure plays the key role. Gray's results show that digital health contributes to climate change through the production and disposal of health wearables and medical devices and through the energy consumption of data and telecommunication centers. Additionally, the energy required for data processing in AI and ML adds to the overall environmental impact. On the other hand, digital health can mitigate climate change through the expansion of virtual care, along with health data science, and analyzing patient data to provide more accurate clinical decisions and reduce overdiagnosis.

### Findings in the literature falling outside the review search

#### Diagnostics, overdiagnosis, and self-tracking technologies

Many new diagnostic methods rely on digitalization and have the potential to decrease the carbon footprint of diagnoses. For example, the carbon footprint of the point of care (PoC) of the C-reactive protein (CRP) test was only 0**·**4% of a whole blood test.^41^ For most tests, the major source of impact was sample collection consumables (swabs, gloves, vacutainer holders and collection tubes, specimen bags), and PoC measurements typically generated less waste. Moreover, PoC measurements promoted decentralization, thereby reducing the need for hospital or laboratory visits.^42^ The need for sample transportation was reduced or eliminated, and rapid results could reduce the need for repeated visits and additional tests, thereby reducing the overall carbon footprint.

Another example of efforts to reduce the GHG emissions of diagnostics were efforts involving magnetic resonance imaging (MRI). These included optimized energy use during active scanning (optimized by adjusting scanner settings, sequences, and protocol lengths) and leveraging AI applications to enhance sustainability in MRI practices.^43^

From an environmental perspective, diagnostic excellence—characterized by accurate and timely diagnoses—was crucial.^44^ For instance, fewer downstream tests and treatments due to early diagnosis could result in reduced carbon footprints.^45–47^

But the widespread availability of diagnostic tests and tools had also resulted in increased healthcare utilization and the overuse of medical services, often without providing any tangible health benefits to individuals.^48^ While the clinical aspects of overuse were often emphasized, its climate impacts remained underappreciated.^49^ Overtesting and overdiagnosis contributed to climate change by generating unnecessary traveling, emissions, and waste. An Australian study^50^ estimated that the carbon footprint of unnecessary vitamin D tests ranged from 28**·**6 t CO_2_e (the base case) to 42**·**0 t CO_2_e (the sensitivity case). Barratt et al.^51^ estimated that the elimination of low value and harmful care—estimated to account for 40% of clinical care—would lead to a 10 Mt CO_2_e emission reduction in Australian healthcare.

Overdiagnosis, both overdetection and overdefinition, have become a common problem in modern healthcare,^52^ especially with widespread, uncontrolled access to diagnostic technologies. For instance, overdiagnosis was considered for several cancer types (prostate, breast, and thyroid cancer; skin melanoma), and possible negative effects related to increased carbon emissions were considered by Barratt and McGain,^53^ although not quantifying the carbon footprints.

Digitalization also enables the rise of self-tracking technologies such as smart watches, rings, and activity trackers. The wearables market has seen significant growth in recent years with typical annual device shipments exceeding over 500 million units globally^54^ and a projected market size exceeding 180 billion by 2030.^55^ While these monitoring tools hold promise in the early detection of disease—encouraging behavioral changes that can prevent disease, enhancing treatment outcomes, and lowering overall healthcare resource consumption—the production and disposal of wearable devices can negatively impact on the environment through heightened energy consumption, material usage, and waste generation.^56^

Estimating the true impact of self-tracking is challenging due to the limited research that reviews, classifies, and synthesizes the state of the art in this area.^57^ There is, however, a growing body of research analyzing the effectiveness of self-tracking and remote monitoring in managing chronic diseases, suggesting they have potential to improve healthcare and reduce the resources used. For example, Bashi et al.^58^ reviewed 10 systematic reviews and found that the remote monitoring of metrics (like blood pressure, heart rate, weight, and electrocardiogram metrics) significantly reduced mortality and rehospitalization rates in patients with chronic heart failure. If the self-tracking devices used for chronic disease monitoring can reduce hospitalizations, they may also help reducing the carbon footprint that results from patient treatments.

#### Artificial intelligence

AI has already had, and will continue to have, a major impact on and role in medicine and healthcare.^59,60^ This is attributed to the increasing awareness and concern regarding the energy and environmental costs of using AI.^22,61–64^ A survey of 4260 AI researchers showed that 57% of the researchers believe that AI could have a positive impact on people's lives through improving access to healthcare. On the other hand, 21% of the AI researchers thought that AI could have a negative impact on people's lives through reducing access to in-person healthcare.^65^ Some of the recent advancements correlate with the training and use of ever larger ML models, like Google's Med-Gemini model^66^ and OpenAI's GPT models.^67^ However, training and using such large models were shown to be very energy demanding.^61^ For example, the GHG emissions for just training the GPT-4 model were estimated to be about 6912 tons of CO_2_e.

Despite the trend of training and utilizing increasingly larger ML models, it is important to note that smaller, and possibly less complex, models can perform certain tasks just as well as some of the more complex models and with far less computational costs and emissions.^68^ This highlights that AI developers should be aware of some ethical issues.^62^ It is important that people developing AI services have an awareness of the underlying computational costs associated with the various ML architectures/models and that they always aim to optimize the balance between performance, utility, and the computational costs of training and using these models.

Despite the challenges associated with the energy costs and carbon emissions of AI, the use of AI technology in healthcare may also help to reduce carbon emissions. This can happen either through directly assisting and optimizing clinical work, such as through identifying overdiagnosis,^59,69^ or through optimizing the processes, equipment requirements and production, facilities, and transportation connected to healthcare.^22^ Finally, the article title used by Tomlinson et al.^70^ is worth noting: “The carbon emissions of writing and illustrating are lower for AI than for humans.” They argued that the overall carbon footprint of completing certain tasks can be reduced when they are solved with the assistance of AI tools, extending this to numerous possible AI-assisted tasks, such as generating or transcribing clinical notes.

#### Innovations

It is most obvious that innovations have been in crucial roles in the development of the digital products and services described above and that innovation-related literature would offer relevant topics also for the theme of this article, even if the search term digital* is missing. Nowadays many innovations in the healthcare sector strive for low-carbon solutions and use and develop digital products and services. We rise here one article, giving a new topic “monitoring” to our framework: Al Amosh and Khatib^71^ found the importance of integrating monitoring systems into innovation strategies to enhance accountability and achieve long-term emissions reductions in the healthcare sector.

## Discussion: digitalization's development and carbon footprint in the healthcare system

### A decrease in carbon footprint due to digital healthcare solutions

In the digitalization cases of our review, the carbon footprint of the virtual consultations ranged between 0·005 and 3 kgCO2e, while the in-person healthcare clinic visits ranged between 0·57 and 178 kgCO2e depending on the mode of transport used, the difference in carbon footprint being 79–99%. Similarly, in-person continuous medical education meetings caused emissions of 254 kgCO2e per participant, while virtual events caused 1 kgCO2e per participant.^37^ However as noted earlier, the transparency of carbon footprint analyses was often low, and the evidence for digitalization decreasing climate impacts was limited. More emphasis is needed on using standardized and transparent carbon footprint methods and reporting. See more description of the carbon footprint values in Supplemental Table S3 (Appendix D).

#### Framework

It is important to define what digitalization is and to consider whether the reviewed papers accurately represent this concept. In the introduction, digitalization was described as the deployment of digital ICTs. The included articles indeed dealt with appliances and related software that measure and process data and transmit information. Although the broad definition of *digitalization* opens many possibilities and can even lead to misunderstandings regarding its subtopics, this was not the case for the reviewed articles.

**Figure 2.**
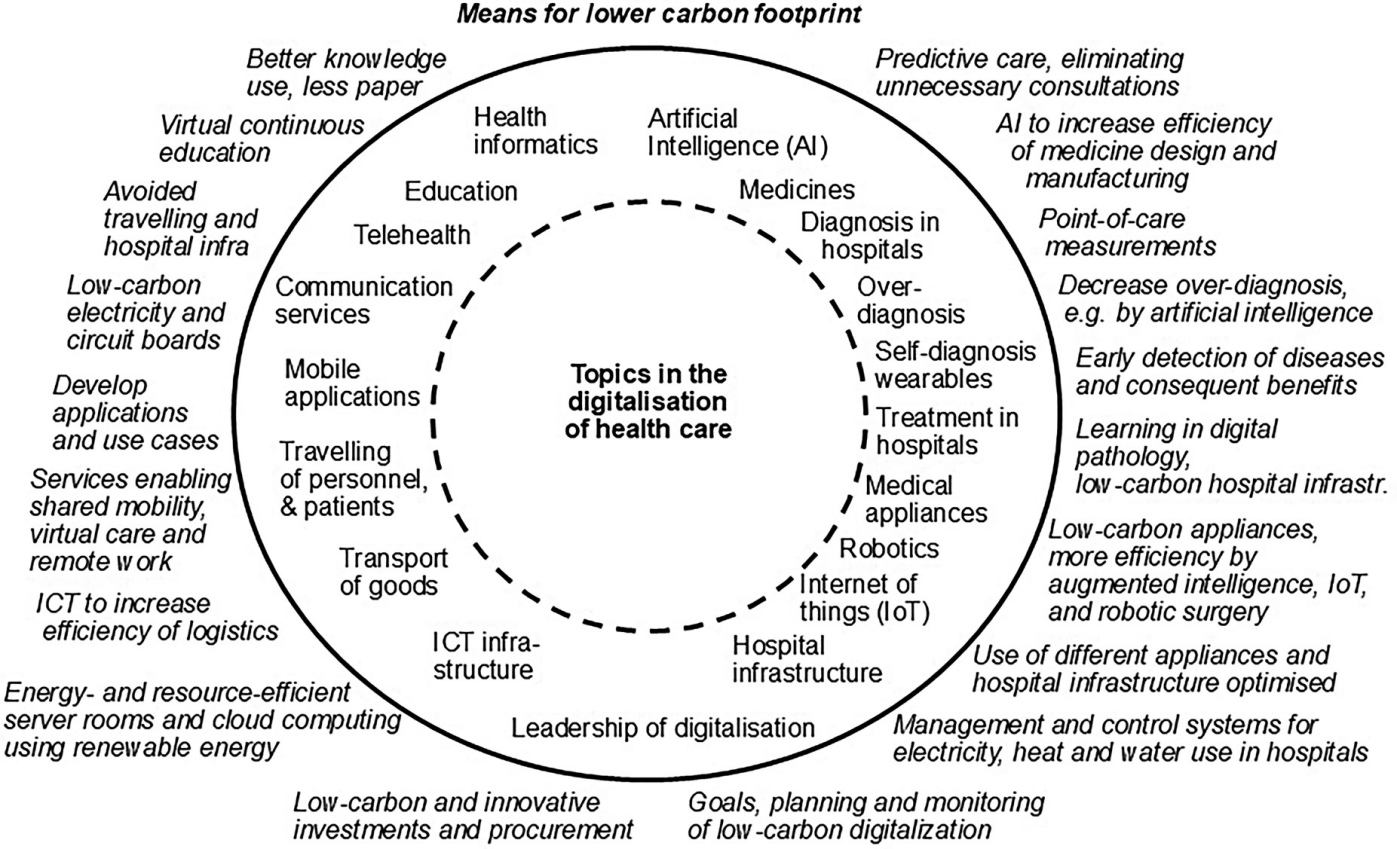
A framework for the digitalization-related topics and means for reaching a lower carbon footprint in healthcare. The topics are inside the circle, and the identified means for reaching lower carbon footprint (i.e. climate change mitigation measures) are outside the circle. The means are at about the same level as the topics, that is, topics and related means are close to each other in the figure.

To start the drawing of the framework, we first identified areas and functions of healthcare where digitalization may play a major role for the carbon footprint. They are seen in Figure 2 and in more detail in Supplemental Table S3 (see Appendix D).

Digitalization affects almost all, if not all, areas of healthcare and has potential for a major decrease in the carbon footprint. It affects the storing of information (*health informatics, ICT infrastructure* in Figure 2). It enables the more efficient processing of information (*AI or augmented intelligence, ICT infrastructure*) and, in this way, improves efficiency in healthcare by improving existing systems and helping create new production methods, products, and services (*diagnosis; self-diagnosis using wearables; treatments in hospitals; medical appliances; medicines manufacturing; the traveling of personnel, patients, visitors; the transport of goods; hospital building infrastructure*). Digitalization increases the transmitting units of information (*health informatics, education, telehealth and virtual care, digital health communication services, mobile applications*) and enables innovating new types of machinery (*robotics*) and connections or networks between separate appliances and the hospital infrastructure (*the IoT*)*.* However, increased possibilities are not always good for efficiency and the carbon footprint (e.g. due to *overdiagnosis*).

Finally, *leadership*, *planning* and *monitoring* are crucial for the operation, further development, and follow-up of the goals of the whole low-carbon digital system (like in Figure 2).

#### Action, foreground, and background

One could say that ICT appliances—especially the electronics in them, the software that regulates their functioning, and the electricity that keeps them functioning—lie at the very heart of digitalization. However, the personnel in healthcare, as well as the patients, see digitalization as the services produced by the medical appliances and automatically adjusted building environments (i.e. digital solutions adjusting warm and light spaces for operations and rest).

**Figure 3.**
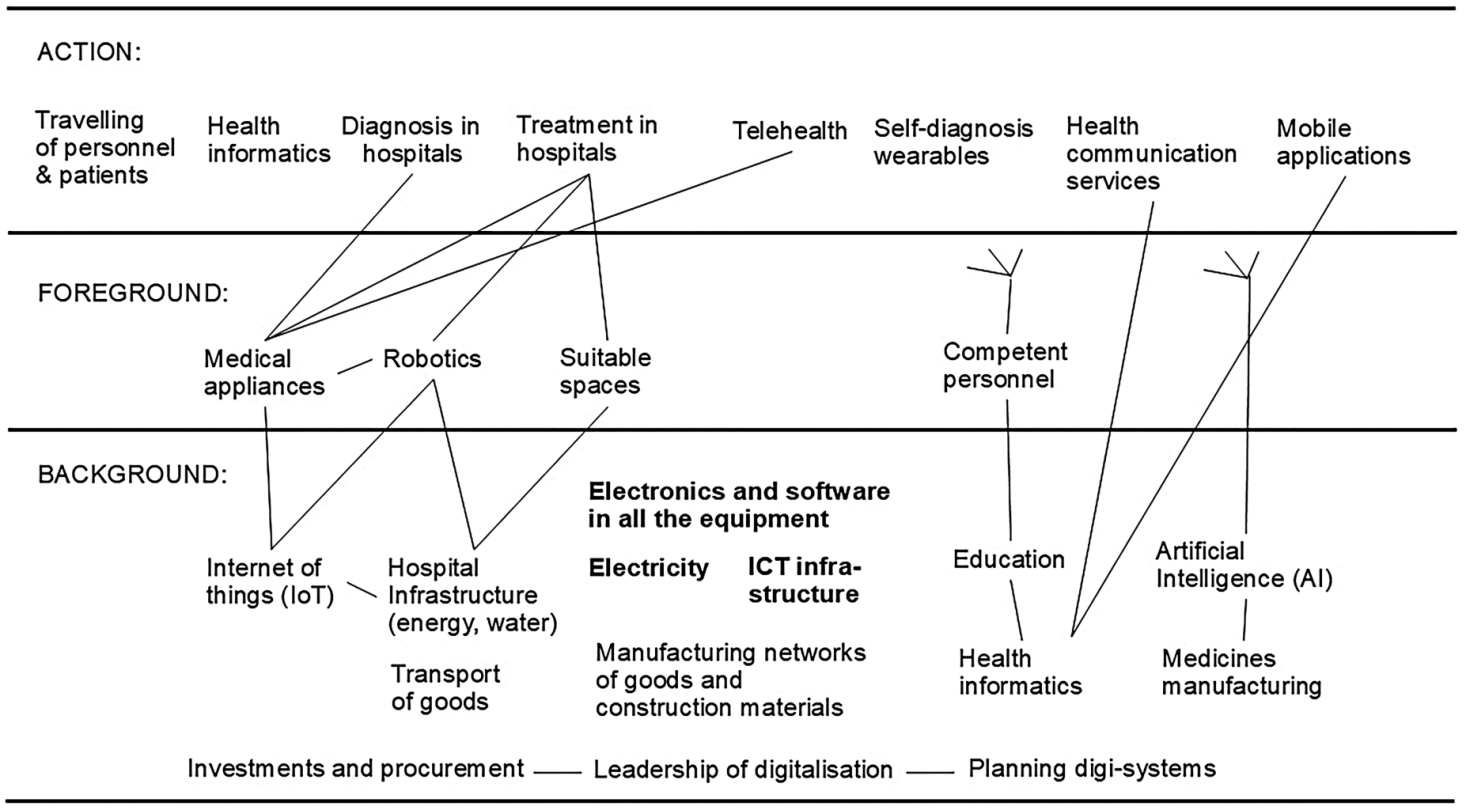
Digitalization topics divided to “action,” “foreground,” and “background.” Some linkages between these are shown by lines.

Building on the ideas above, the digitalization topics can be divided into “action,” “foreground,” and “background” (see Figure 3). “Action” includes *diagnoses* and *treatments* in hospitals and healthcare centers, *health informatics* when patients register into them, and the *traveling of personnel, patients, and visitors.* Outside hospitals and health centers, “action” includes *telehealth (virtual care), self-diagnosis using wearables, digital health communication services,* and *mobile applications.*

“Foreground” includes the equipment and services that the patients see (but do not actively interact with) when being diagnosed and treated: *medical appliances* and *robotics.*

“Background” includes many crucial topics that patients do not see: *health informatics, education (of the personnel), AI, medicines manufacturing, IoT, transport of goods, hospital building infrastructure,* and *ICT infrastructure*. An additional important topic in the background is also the *manufacturing networks of goods and construction materials*, with the digitalization solutions involved in this increasing the efficiency of manufacturing and also affecting the carbon footprints of the goods and materials. *Leadership* and *planning* steer the operation and the further development. In order to get low-carbon solutions (goods, services, and facilities), the *investments* and *procurement* of low-carbon solutions should be present in the strategy of the healthcare sector and each organization, and the actual procurement needs to take into account the investments’ and procured products’ carbon footprint.

Two additional topics are evident and need to be foregrounded. They are properties of healthcare and, thus, seen and experienced by the patients: *suitable spaces* and *competent personnel*. They are the results of an efficient background. Warm, well-lit, and clean spaces for operations and resting places for patients are the results of *hospital building infrastructure*. *Competent personnel* are the result of efficient *education*.

Some of the linkages between “background,” “foreground,” and “action” are shown by the lines in Figure 3. However, *electronics and software*, *electricity, ICT infrastructure, manufacturing networks,* and *competent personnel* have so many linkages that it was not meaningful to show all the linkages by drawing the lines. *AI* has the potential to be present in all the software involved in the foreground and background, and thus, it can then also affect all the actions.

An alternative division of key factors related to the digital health technologies has been proposed by Alami et al.^72^ They categorized the factors constraining or enabling the integration of the environmental impacts on digital health technologies into the following categories: innovation factors, organizational factors, and external factors. As they remarked, the categories highlight the complexity of environmental measures in the healthcare sector.

### Limitations

The scoping review may exclude relevant studies that are not framed by digitalization or digitality. These may include, for example, studies specific to certain fields of healthcare or focusing on innovations. The issue was partly addressed by considering some emerging topics of healthcare in addition to the literature review. The actual review only considered articles published before 1 March 2024 in English, which may have resulted in the exclusion of relevant later studies and studies in other languages.

## Conclusions

Digitalization plays a critical role in the changing healthcare. Depending on how digital solutions are implemented, for example, the carbon footprint of the consumed electricity and of the procured electronic devices and materials, they can either reduce or increase the carbon footprint, highlighting the importance of how healthcare systems approach and manage technological advancements. However, the transparency of carbon footprint analyses was often low, system boundaries in the analyses were often different, and the evidence for digitalization decreasing or increasing climate impacts was limited.

Knowledge about the GHG emissions associated with various healthcare functions is important, but it is equally critical to explore how digitalization can help reduce these emissions because digitalization offers the means to do so and digitalization's development goes on even without a focus on climate change mitigation. Designing the change to be geared toward low-carbon solutions and more broadly sustainable solutions makes it possible to steer low-carbon and possible climate neutrality development paths.

As leadership and planning were almost totally missing from the studied articles, it seems that the focus has been on concrete things, like energy and actual services and products, especially in regard to telehealth. More emphasis is needed on having a system-based approach and transparent carbon footprint analyses. To be influential, healthcare organizations and the entire healthcare sector must set strategical goals and lead the digitalization efforts toward a radically lower carbon footprint.

## Supplemental Material

sj-docx-1-dhj-10.1177_20552076251364666 - Supplemental material for The climate impacts of healthcare digitalization: A scoping review Supplemental material, sj-docx-1-dhj-10.1177_20552076251364666 for The climate impacts of healthcare digitalization: A scoping review by Ari Nissinen, Atte Pitkänen, Anton Barchuk, Aida Hosseinian, Annika Johansson, Matti Kaisti, Jaakko Karvonen, Pekka Marttinen, Hans Moen, Emilia Peltola, Laura Sokka and Kari A. O. Tikkinen in DIGITAL HEALTH
