# Supplementary material for: The climate impacts of healthcare digitalization: A scoping review
Source: Digit Health. 2025 Aug 25;11:20552076251364666. doi: 10.1177/20552076251364666 (PMC12378540; doi:10.1177/20552076251364666)
Supplement: sj-docx-1-dhj-10.1177_20552076251364666 - Supplemental material for The climate impacts of healthcare digitalization: A scoping review [file sj-docx-1-dhj-10.1177_20552076251364666.docx]

**Supplementary Appendix**

Contents

[Appendix A. PRISMA-ScR Checklist 1](#_Toc202135980)

[Appendix B. Search strategy 3](#_Toc202135981)

[Appendix C. Summary of articles included in the scoping review 4](#_Toc202135982)

[Appendix D. The areas and functions of the healthcare in which digitalization may play a major role regarding the carbon footprint 11](#_Toc202135983)

# Appendix A. PRISMA-ScR Checklist

**Preferred Reporting Items for Systematic reviews and Meta-Analyses extension for Scoping Reviews (PRISMA-ScR) Checklist**

| **SECTION** | **ITEM** | **PRISMA-ScR CHECKLIST ITEM** | **REPORTED ON PAGE #** |
| --- | --- | --- | --- |
| **TITLE** | | | |
| Title | 1 | Identify the report as a scoping review. | 1 |
| **ABSTRACT** | | | |
| Structured summary | 2 | Provide a structured summary that includes (as applicable): background, objectives, eligibility criteria, sources of evidence, charting methods, results, and conclusions that relate to the review questions and objectives. | 1 |
| **INTRODUCTION** | | | |
| Rationale | 3 | Describe the rationale for the review in the context of what is already known. Explain why the review questions/objectives lend themselves to a scoping review approach. | 1-2 |
| Objectives | 4 | Provide an explicit statement of the questions and objectives being addressed with reference to their key elements (e.g., population or participants, concepts, and context) or other relevant key elements used to conceptualize the review questions and/or objectives. | 1-2 |
| **METHODS** | | | |
| Protocol and registration | 5 | Indicate whether a review protocol exists; state if and where it can be accessed (e.g., a Web address); and if available, provide registration information, including the registration number. | 2 |
| Eligibility criteria | 6 | Specify characteristics of the sources of evidence used as eligibility criteria (e.g., years considered, language, and publication status), and provide a rationale. | 2-3 |
| Information sources* | 7 | Describe all information sources in the search (e.g., databases with dates of coverage and contact with authors to identify additional sources), as well as the date the most recent search was executed. | 2 |
| Search | 8 | Present the full electronic search strategy for at least 1 database, including any limits used, such that it could be repeated. | Appendix B |
| Selection of sources of evidence† | 9 | State the process for selecting sources of evidence (i.e., screening and eligibility) included in the scoping review. | 2-3 |
| Data charting process‡ | 10 | Describe the methods of charting data from the included sources of evidence (e.g., calibrated forms or forms that have been tested by the team before their use, and whether data charting was done independently or in duplicate) and any processes for obtaining and confirming data from investigators. | 3 |
| Data items | 11 | List and define all variables for which data were sought and any assumptions and simplifications made. | 3 |
| Critical appraisal of individual sources of evidence§ | 12 | If done, provide a rationale for conducting a critical appraisal of included sources of evidence; describe the methods used and how this information was used in any data synthesis (if appropriate). | N/A |
| Synthesis of results | 13 | Describe the methods of handling and summarizing the data that were charted. | 3 |
| **RESULTS** | | | |
| Selection of sources of evidence | 14 | Give numbers of sources of evidence screened, assessed for eligibility, and included in the review, with reasons for exclusions at each stage, ideally using a flow diagram. | 3 |
| Characteristics of sources of evidence | 15 | For each source of evidence, present characteristics for which data were charted and provide the citations. | Appendix C |
| Critical appraisal within sources of evidence | 16 | If done, present data on critical appraisal of included sources of evidence (see item 12). | N/A |
| Results of individual sources of evidence | 17 | For each included source of evidence, present the relevant data that were charted that relate to the review questions and objectives. | 3-6 |
| Synthesis of results | 18 | Summarize and/or present the charting results as they relate to the review questions and objectives. | Appendix D |
| **DISCUSSION** | | | |
| Summary of evidence | 19 | Summarize the main results (including an overview of concepts, themes, and types of evidence available), link to the review questions and objectives, and consider the relevance to key groups. | 8-10 |
| Limitations | 20 | Discuss the limitations of the scoping review process. | 10 |
| Conclusions | 21 | Provide a general interpretation of the results with respect to the review questions and objectives, as well as potential implications and/or next steps. | 10-11 |
| **FUNDING** | | | |
| Funding | 22 | Describe sources of funding for the included sources of evidence, as well as sources of funding for the scoping review. Describe the role of the funders of the scoping review. | 11 |

JBI = Joanna Briggs Institute; PRISMA-ScR = Preferred Reporting Items for Systematic reviews and Meta-Analyses extension for Scoping Reviews.

* Where *sources of evidence* (see second footnote) are compiled from, such as bibliographic databases, social media platforms, and Web sites.

† A more inclusive/heterogeneous term used to account for the different types of evidence or data sources (e.g., quantitative and/or qualitative research, expert opinion, and policy documents) that may be eligible in a scoping review as opposed to only studies. This is not to be confused with *information sources* (see first footnote).

‡ The frameworks by Arksey and O’Malley (6) and Levac and colleagues (7) and the JBI guidance (4, 5) refer to the process of data extraction in a scoping review as data charting*.*

§ The process of systematically examining research evidence to assess its validity, results, and relevance before using it to inform a decision. This term is used for items 12 and 19 instead of "risk of bias" (which is more applicable to systematic reviews of interventions) to include and acknowledge the various sources of evidence that may be used in a scoping review (e.g., quantitative and/or qualitative research, expert opinion, and policy document).

*From:* Tricco AC, Lillie E, Zarin W, O'Brien KK, Colquhoun H, Levac D, et al. PRISMA Extension for Scoping Reviews (PRISMAScR): Checklist and Explanation. Ann Intern Med. 2018;169:467–473. [doi: 10.7326/M18-0850](http://annals.org/aim/fullarticle/2700389/prisma-extension-scoping-reviews-prisma-scr-checklist-explanation).

# Appendix B. Search strategy

**Supplementary Table S1: Search strategy**

|  | **Search terms** | **Database (fields) searched** |
| --- | --- | --- |
| Search 1 | digital* AND (health* OR hospital* OR patient* OR medic* OR pharmaceutical*) AND (“carbon footprint*” OR “carbon emission*” OR “CO2 emission*” OR “greenhouse gas emission*” OR "environmental impact*" OR "life cycle assessment*") | Web of Science (title, abstract, keywords) |
|  | digital*[Title/Abstract] AND (health*[Title/Abstract] OR hospital*[Title/Abstract] OR patient*[Title/Abstract] OR medic*[Title/Abstract] OR pharmaceutical*[Title/Abstract]) AND ("carbon footprint*"[Title/Abstract] OR "carbon emission*"[Title/Abstract] OR "CO2 emission*"[Title/Abstract] OR "greenhouse gas emission*"[Title/Abstract] OR "environmental impact*"[Title/Abstract] OR "life cycle assessment*"[Title/Abstract]) | PubMed (title, abstract) |
| Search 2 | ((digital* AND (health* OR hospital* OR patient* OR medic* OR pharmaceutical*)) OR (telemedicine* OR telehealth* OR telecare* OR “virtual consulting*” OR “virtual care*”)) AND (“carbon footprint*” OR “carbon emission*” OR “CO2 emission*” OR “greenhouse gas emission*” OR "environmental impact*" OR "life cycle assessment*") | Web of Science (title, abstract, keywords) |
|  | ((digital*[Title/Abstract] AND (health*[Title/Abstract] OR hospital*[Title/Abstract] OR patient*[Title/Abstract] OR medic*[Title/Abstract] OR pharmaceutical*[Title/Abstract])) OR (telemedicine*[Title/Abstract] OR telehealth*[Title/Abstract] OR telecare*[Title/Abstract] OR "virtual consulting*"[Title/Abstract] OR "virtual care*"[Title/Abstract])) AND ("carbon footprint*"[Title/Abstract] OR "carbon emission*"[Title/Abstract] OR "CO2 emission*"[Title/Abstract] OR "greenhouse gas emission*"[Title/Abstract] OR "environmental impact*"[Title/Abstract] OR "life cycle assessment*"[Title/Abstract]) | PubMed (title, abstract) |

# Appendix C. Summary of articles included in the scoping review

***Supplementary Table S2:* Summary of articles included in the scoping review.**

| **Original research articles** | | | | | | | |
| --- | --- | --- | --- | --- | --- | --- | --- |
| **Authors** | **Year** | **Title** | **Study design** | **Aims/objectives** | **Aspect of healthcare digitalization** | **System boundaries (research articles)** | **Key findings of climate impacts** |
| Elbadawi et al. | 2023 | Energy consumption and carbon footprint of 3D printing in pharmaceutical manufacture | Original research | To investigate the energy consumption of 3D printer types commonly used in producing medicines | 3D printed pharmaceuticals | Standby and printing energy consumption for six different printers. CO2e conversion factor for UK electricity. | The energy consumption values are comparable to conventional tablet production by powder compaction. Reducing printing temperatures could achieve reductions between 5,88-33,3%. |
| Gandhi et al. | 2023 | Impact of virtual continued medical education on carbon footprint and awareness of digital sobriety: A retrospective cross-sectional study among public health professionals in India | Original research | To assess the impact of virtual continuous medical educations on the environment | Virtual vs. in-person continuous medical education | Travel, accommodation, food, certificates, virtual infrastructure and electricity consumption. India | The impact of the virtual event was ca. 1 kg CO2eq./participant while that of physical meetings would be 254 kg CO2eq./participant. Thus, the impact of virtual meetings would be over 99% lower than that of physical meetings, mainly through avoided travel. |
| Kwon et al. | 2024 | The Environmental Impacts of Electronic Medical Records Versus Paper Records at a Large Eye Hospital in India: Life Cycle Assessment Study | Original research | To estimate and compare the environmental emissions associated with paper medical record-keeping and its replacement with electronic medical record system at a high-volume eye care facility in southern India | Electronic medical record system | Life cycle assessment (electronic medical record vs. paper record-keeping system) | Climate impacts of the electronic medical record system would be about 10 times higher than those of the paper-based system. This difference mainly originates from the high emissions of the Indian electricity. If renewable electricity was used, the electronic medical record system would only have about 20% higher climate impacts than the paper-based system. |
| Li et al. | 2023 | Optimizing environmental sustainability in pharmaceutical 3D printing through machine learning | Original research | To investigate the environmental effects of pharmaceutical 3D printing by using Design of Experiments and Machine Learning. More precisely, to assess the energy use in pharmaceutical Fused Deposition Modeling, and to identify several key parameters that contribute to energy consumption, and consequently CO2 emissions | 3D printed pharmaceuticals | Energy consumption. CO2e conversion factor for UK electricity. | Process temperature and other factors are associated with emissions; machine learning can reduce emissions |
| Palmer et al. | 2023 | Optimizing an Adolescent Hybrid Telemedical Mental Health Service Through Staff Scheduling Using Mathematical Programming: Model Development Study | Original research | To develop a mathematical program that schedules mental health-care practitioners and assigns them to either the base of the telehealth care service or to hospitals where staff would be required to assess or treat patients. To find a minimum coverage for staff by satisfying time- and location-dependent demands and to minimize travel distances. | Hybrid telehealth service. | Staff travel. Modelling the possibilities to reduce travel times and the number of staff through telemedicine (video consultation). | Staff travel can be optimized through mathematical modelling leading to lower travel distances and GHG emissions. |
| Tarpani and Gallego-Schmid | 2024 | Environmental impacts of a digital health and well-being service in elderly living schemes | Original research | To examine the life cycle impacts of a digital service implemented in three elderly living schemes (ELSs) located in the United Kingdom (UK) | Digital health service | Life cycle assessment of a digital health communication service. UK | The consumption of electricity was found to be the main contributor to most of the environmental impacts. However, in certain categories, printed circuit boards (PCBs) were the main contributors (freshwater eutrophication, human toxicity non-cancer). |
| Vafaei Sadr et al. | 2024 | Operational greenhouse-gas emissions of deep learning in digital pathology: a modelling study | Original research | To conduct an environmental-sustainability analysis of a theoretical implementation of deep learning in patient-care pathology | Deep learning in pathology | GHG emissions of various deep-learning systems in pathology. Electricity consumption; computational devices. Germany | Widespread use of deep learning in pathology might have considerable global-warming potential.  The substantial variations in GHG emissions between different deep-learning models show the importance of model selection and size.  The reduction of input data and the selection of computationally less demanding models could reduce energy consumption. |

| **Original research articles - telemedicine** | | | | | | | |
| --- | --- | --- | --- | --- | --- | --- | --- |
| **Authors** | **Year** | **Title** | **Study design** | **Aims/objectives** | **Aspect of healthcare digitalization** | **System boundaries (research articles)** | **Key findings of climate impacts** |
| Arndt et al. | 2023 | COVID-19 measures as an opportunity to reduce the environmental footprint in orthopedics and trauma surgery | Original research | To analyse the effect of video consultation on the CO2 emissions during the Covid-19 pandemic in an outpatient clinic of the department of orthopaedics and traumatology surgery at a German university hospital | Telemedicine; CF reduction through video consultations (in an outpatient clinic of the department of orthopedics and traumatology surgery) | Patient travel; Video consultation operative emissions. Germany | CF reduction of video consultations compared to a face-to-face presentation was 97% |
| Bartlett and Keir | 2022 | Calculating the carbon footprint of a Geriatric Medicine clinic before and after COVID-19 | Short report | To estimate the carbon footprint of a Geriatric Medicine clinic, including the effect of virtual consultation and personal protective equipment, in order to inform design of a service that addresses both the health of our patients and our environment | Telemedicine; CF of face-to-face and virtual geriatric clinic consultations | Patient and staff travel; Telecommunications; Personal protective equipment; Water use; Energy use for rooms.  UK | CF of a face-to-face clinic consultation is 4.82 kgCO2e, most of which is patient travel (60%), followed by staff travel (35%) and use of personal protective equipment (3%), while the CF of a virtual consultation is 0.99 kgCO2e, most of which is staff travel (56%), followed by data use (37%). |
| Holmner et al. | 2014 | Carbon Footprint of Telemedicine Solutions Unexplored Opportunity for Reducing Carbon Emissions in the Health Sector | Original research | To evaluate the potential of telemedicine services based on videoconferencing technology to reduce travelling and thus carbon emissions in the healthcare sector | Telemedicine; CF of video appointments vs. care-as-usual scenarios | Patient travel; Videoconferencing equipment; Data and energy usage.  Sweden | Telemedicine appointments resulted in 40-70 times decrease in carbon emissions compared to physical visits; Carbon emissions from a one-hour meeting using a desktop solution are exceeded by the emissions from a car driving as little as a few kilometers |
| King et al. | 2023 | Towards NHS Zero: greener gastroenterology and the impact of virtual clinics on carbon emissions and patient outcomes. A multisite, observational, cross-sectional study. | Original research | To evaluate the impact of telemedicine on emission reduction and patient outcomes in the gastroenterology outpatient setting | Telemedicine; CF reduction through remote consultations in a gastroenterology clinic | Patient travel; Emissions produced by phone calls (all virtual appointments were performed using hospital landline telephones, no videoconferencing services were used).  UK | Estimated reduction of 5.35 kg CO2 per appointment through remote consultations (99.07%). |
| Morcillo Serra et al. | 2022 | Impact on the reduction of CO2 emissions due to the use of telemedicine | Original research | To evaluate the environmental impact of digital health solutions by analysing the net CO2 emissions avoided thanks to the corresponding reduction in patient travel to medical clinics when using a digital consultation or when downloading medical reports instead of travelling in to request printed versions | Telemedicine | Patient travel; Paper and printing; Videoconferencing emissions.  Spain | Telemedicine reduces emissions primarily through reduced travel: an average of 3.057 kg of net CO2 emissions avoided for every digital appointment |
| Penaskovic et al. | 2022 | Telehealth: Reducing Patients’ Greenhouse Gas Emissions at One Academic Psychiatry Department | Original research | To estimate the reduction of patients’ greenhouse gas emissions during the rapid shift to the use of more telehealth by behavioral health clinicians at one academic psychiatry institution | Telemedicine; CF reduction through virtual psychiatry clinic visits | Patient travel; Usage of patients' telemedicine equipment.  USA | 21 kgCO2 emission decrease per visit through telemedicine |
| Schmitz-Grosz et al. | 2023 | A Telemedicine Center Reduces the Comprehensive Carbon Footprint in Primary Care: A Monocenter, Retrospective Study | Original research | To quantify the impact of a telemedicine center on the carbon footprint of primary care consultations | Telemedicine center’s impact on the CF of primary care consultations | Prevented and provoked patient journeys (in-person visits resulting from telemedical consultation); Telemedicine center infrastructure.  Switzerland | 0.57 kgCO2e saving per telemedical consultation. |
| Sillcox et al. | 2023 | The environmental impact of surgical telemedicine: life cycle assessment of virtual vs. inperson preoperative evaluations for benign foregut disease | Original research | To quantify the current usage and carbon footprint of telemedicine in a general surgery subspecialty clinic | Telemedicine; CF of virtual vs. inperson preoperative evaluations | Patient travel: Materials and durable medical equipment used during an in-person visit; Equipment and network data usage related to telemedicine encounters. The total environmental impact of the manufacturing and end-of -life disposal of the devices to determine the per-hour environmental impact of computer usage. USA | Telemedicine GHG emissions ranged from 2.26 to 2.99 kgCO2e per visit depending on the device used. An in-person visit resulted in GHG emissions of 38.22 kgCO2e with car travel and 39.61 kgCO2e with air travel. |
| Thiel et al. | 2023 | Telemedicine and the environment: life cycle environmental emissions from in-person and virtual clinic visits | Original research | To determine the environmental emissions associated with in-person and virtual clinic visit | Telemedicine; CF of virtual vs. in-person clinical visit | Patient travel; HVAC and lighting energy; Supplies used; Waste generated; Video or phone call (electricity used by patient and staff). USA | On average, an in-person visit emitted an estimated 20 kg CO2e, while a phone-based virtual visit emitted only 0.02 kg CO2e, and a video visit emitted 0.04 kg CO2e. |

| **Review articles** | | | | | | | |
| --- | --- | --- | --- | --- | --- | --- | --- |
| **Authors** | **Year** | **Title** | **Study design** | **Aims/objectives** | **Aspect of healthcare digitalization** | **System boundaries (research articles)** | **Key findings of climate impacts** |
| Das and Chandra | 2023 | A survey on artificial intelligence for reducing the climate footprint in healthcare | Narrative review approach | To review the potential of AI in healthcare for lowering emission rates | AI in healthcare | NA | Data-driven and well-optimized AI can reduce healthcare's carbon footprint through several mechanisms |
| Lange et al. | 2022 | A Transparency Checklist for Carbon Footprint Calculations Applied within a Systematic Review of Virtual Care Interventions | Systematic review | To develop a transparency catalogue for reporting carbon footprint calculations, to compare results, and to assess the transparency (reporting quality) of the current evidence of virtual care intervention | Virtual care | NA | Literature shows carbon savings through virtual care; but low transparency in calculations provides only weak evidence |
| Lokmic-Tomkins et al. | 2022 | Assessing the carbon footprint of digital health interventions: a scoping review | Scoping review | To survey the evidence on available environmental assessment frameworks, methods, and tools to evaluate the carbon footprint of digital health interventions for environmentally sustainable healthcare | Digital health interventions | NA | Literature mostly focuses on telehealth emissions; systems-based approach is needed |
| Pickard Strange et al. | 2023 | The Role of Virtual Consulting in Developing Environmentally Sustainable Health Care: Systematic Literature Review | Systematic review | What is the impact of virtual consulting on environmental sustainability in healthcare? What can we learn from current evaluations that can inform future reductions in carbon emissions? | Virtual consulting | NA | There is overwhelming evidence that virtual consulting can reduce healthcare carbon emissions, largely through reducing travel related to in-person appointments |
| Purohit et al. | 2021 | Does telemedicine reduce the carbon footprint of healthcare? A systematic review | Systematic review | To conduct a systematic review of the evidence on the carbon footprint of telemedicine | Telemedicine | NA | CF savings in literature range between 0.70-372 kgCO2e per consultation, but the values are highly context specific. Telemedicine systems themselves produce low emissions compared to the emissions saved from travel reductions. A strong relationship was found between CF savings and travel distance savings. |
| Rahimi-Ardabili et al. | 2022 | Digital health for climate change mitigation and response: a scoping review | Scoping review | To explore recent work on digital health responses and mitigation approaches to climate change | Digital health (& climate change mitigation) | NA | Literature mostly focuses on telehealth emissions; systems-based approach is needed |
| Ravindrane and Patel | 2022 | The environmental impacts of telemedicine in place of face-to-face patient care: a systematic review | Systematic review | To determine whether telemedicine has environmental benefits and to quantify these benefits. To identify whether the environmental benefits of telemedicine vary by the form of telemedicine used or the health service setting. | Telemedicine | NA | All 14 reviewed studies reported CF reductions through telemedicine vs. face-to-face consultations. Three of the studies accounted for the CF of telemedicine equipment. The studies included in the review were found poor in their quality. |
| Rodler et al. | 2023 | The Impact of Telemedicine in Reducing the Carbon Footprint in Health Care: A Systematic Review and Cumulative Analysis of 68 million Clinical Consultations | Systematic review and cumulative analysis | To investigate the reduction in carbon footprint for patients utilizing telemedicine | Telemedicine | NA | Telemedicine reduces the CF of the healthcare sector. |
| Samuel and Lucassen | 2022 | The environmental sustainability of data-driven health research: A scoping review | Scoping review | To conduct a scoping review of how the environmental impacts of data storage and processing during Data-Driven and Artificial Intelligence health-related research are being discussed in the academic literature | Data-Driven and Artificial Intelligence health research | NA | Scoping review about the environmental impacts of data storage and processing during Data-Driven and Artificial Intelligence health related research |
| Schmidt and Bohnet-Joschko | 2022 | Planetary Health and Hospitals’ Contribution—A Scoping Review | Scoping review | To provide the state of research on hospitals’ carbon footprint and to determine their contribution to mitigating emissions | Hospitals' carbon footprint | NA | Digital transformation, in the form of telemedicine, is a key factor in implementing climate actions in hospitals |
| Yellowlees | 2022 | Climate Change Impacts on Mental Health Will Lead to Increased Digitization of Mental Health Care | Review | To review the evidence for the impact of climate change on the mental health of individuals and communities, and to address the literature on the importance of digital systems in reducing carbon emissions | Digitalization of mental healthcare | NA | Telepsychiatry can reduce carbon emissions by reducing patient and provider travel |

| **Special articles** | | | | | | | |
| --- | --- | --- | --- | --- | --- | --- | --- |
| **Authors** | **Year** | **Title** | **Study design** | **Aims/objectives** | **Aspect of healthcare digitalization** | **System boundaries (research articles)** | **Key findings of climate impacts** |
| Fragão-Marques and Ozben | 2023 | Digital transformation and sustainability in healthcare and clinical laboratories | Opinion | To explore current evidence on digital technologies’ impact in healthcare and clinical laboratories | Digital health technologies | NA | Telehealth can reduce emissions; digitalization requires adequate planning |
| Gray | 2022 | Climate Change, Human Health, and Health Informatics: A New View of Connected and Sustainable Digital Health | Mini review | To briefly review examples of recent international work on two fronts: to consider how health informatics can reduce the carbon footprint of healthcare, and to consider how it can integrate new kinds of data for insights into the human health impacts of climate change | Health informatics | NA | Low-carbon health IT infrastructure plays the key role to achieve emission reductions |
| Lokmic-Tomkins et al. | 2023 | Designing digital health applications for climate change mitigation and adaptation | Perspective | Perspectives on how can low carbon models of care be achieved through digital health, and what are the implications for digital health service design and delivery? How can digital solutions be adapted on the principles of sustainability, ecology, and equity in order to effectively address the impacts of climate change on health? | Digital health (& low-carbon healthcare) | NA | Digital health can reduce emissions but also contributes to climate change |
| Thompson | 2021 | The Environmental Impacts of Digital Health | Commentary | To draw attention to the environmental impacts of digital health devices and communication networks, as well as the data produced by digital health activities | Digital health | NA | Harmful environmental impacts of digital health have been overlooked |
| Wolf et al. | 2022 | Potential reduction in healthcare carbon footprint by autonomous artificial intelligence | Commentary article | To estimate the potential GHG emission reduction, if any, that can be achieved by the deployment of autonomous AI for point of care diagnostics | Autonomous AI encounter vs. in-person encounter | NA | The marginal GHG emission generated by one additional diabetic eye exam is examined. The use of a specific autonomous AI can lead to an 80% reduction in GHG emissions in the studied state and thereby compensate for increasing carbon emissions attributed to the IT sector.  Autonomous AI can provide significant emissions reductions in healthcare through reduced travel and facility needs. |

# Appendix D. The areas and functions of the healthcare in which digitalization may play a major role regarding the carbon footprint

Regarding the carbon footprint values presented below, for most cases we recommend only considering the carbon footprints as indicative of the order of magnitude rather than as exact carbon footprint values. In agreement with the work of Lange et al.^1^, we see that the analyses usually have restricted system boundaries or do not cover all the resource flows necessary to determine a carbon footprint according to the standard ISO 14067^2^. Consequently, the carbon footprint values presented should be considered as minimum estimates for each function or service. Nonetheless, they serve to illustrate the scale of carbon footprint across various areas of healthcare.

We use the term *low-carbon* instead of terms like ‘zero-carbon’ and ‘net-zero’. While societies committed to the Paris Agreement to achieve net zero emissions by 2050, in order to limit global warming to 1.5 degrees Celsius^3^, and while for various products and services the terms like *net-zero* or *zero-carbon* are commonly used, we prefer the term *low-carbon* in this context. We chose this terminology because almost every product or service contribute to climate warming during its life cycle, i.e., almost every product has a positive value for the carbon footprint, and net-zero requires compensation efforts. It is important to first decrease life-cycle emissions and only then focus on compensating for the residual emissions, and for this reason *low-carbon* products are important.

***Supplementary Table S3:* The areas and functions of the healthcare in which digitalization may play a major role regarding the carbon footprint**

| **The areas and functions of digital healthcare** | **References** | **Greenhouse gas emission change** | |
| --- | --- | --- | --- |
|  |  | **The mechanisms for an emission decrease (D) and increase (I), and the possibility for two-way impacts (2W).** | **The estimates**  **of the carbon footprint;**  **g = gCO_2_e,**  **kg = kgCO_2_e,**  **% = relative decrease in kgCO_2_e as a %** |
| Health informatics: Electronic health records, clinical decision support, data-driven health research,  e-prescriptions, building awareness of climate issues | Gray 2022;  Kwon et al. 2024;  Lokmic-Tomkins et al. 2022;  Lokmic-Tomkins et al. 2023;  Rahimi-Ardabili et al. 2022;  Samuel and Lucassen 2022 | D: More knowledge by health data science, e.g. CF analyses and LCAs of treatment options. Less paper. Increasing the efficiency of the codes to analyse the data.  I: Added emissions of IT infrastructure use, and of electricity. Growing storage and processing of health-related data. Energy-hungry algorithms for data analyses. | Kwon et al:  Electronic medical record with Indian electricity grid 0.361 kg per patient visit, with renewable electricity 0.046 kg. Paper record 0.037 kg. |
| Artificial intelligence, augmented intelligence | Das and Chandra 2023;  Gray 2022;  Lokmic-Tomkins et al. 2023;  Samuel and Lucassen 2022;  Wolf et al. 2022 | D: Predictive maintenance, eliminating unnecessary consultations. For data analysis, tiny AI models with reduced energy requitements during computation. Reduced healthcare facility needs and energy consumption, and reduced travel.  I: Added emissions of IT infrastructure use. | Das and Chandra:  For different industries and services decrease in 3-5 years 15-18 %.  Wolf et al:  Diabetic eye exam 0.02-0.2 g, decrease 80 % using autonomous AI. |
| Education | Gandhi et al. 2023 | D: Virtual continuous medical education (CME) instead of in-person. India. | Gandhi et al:  in-person CME 254 kg per person,  virtual 1 kg.  99,7 %. |
| Telehealth,  virtual care | Bartlett and Keir 2022;  Holmner et al. 2014;  King 2023;  Morcillo Serra et al. 2022;  Penaskovic et al. 2022; Schmitz-Grosz et al. 2023;  Sillcox et al. 2023;  Thiel et al. 2023  Reviews:  Lange et al. 2022;  Pickard Strange et al. 2023;  Purohit et al. 2021;  Rodler et al. 2023; Ravindrane and Patel 2022;  Yellowlees 2022 | D: Avoided travel of patients, and of personnel. Avoided hospital infra. Mathematical modelling for the optimization of staff travel in a hybrid telehealth service.  I: Added emissions from the use of remote telemedicine equipment, in many articles this assessed to have a low impact.    Most studies lack systems-based approach and standardized methodologies (Lange, Lokmic-Tomkins, Rahimi) and e.g. do not present system boundaries and flows of resources (Lange). Lange developed a transparency score for the carbon footprint analyses, and it had a low average value: 38 %. | Bartlett and Keir:  face-to-face consultation 4.8 kg, (virtual 1.0 kg).  Holmner et al:90–178 kg (2.5 kg).  King:1.54 kg (0.005 kg).  Penaskovic et al: 21.6 kg (0.13 kg).  Schmitz-Grosz et al: 0.57 kg (<0.01 kg).  Sillcox et al: 39 kg, (2.3-3.0 kg).  Thiel et al: 20 kg (0.02-0.04 kg).  All studies show decrease in CF through telemedicine, range being 79-99.9%.  Lange: average decrease 148 kg per patient (11 studies), 128 kg per consultation (6 studies). |
| Digital health communication services | Tarpani and Gallego-Schmid 2024 | D: For three elderly living schemes, largest CF contribution from electricity and circuit boards in appliances, apply low-carbon solutions for them, also extending the lifetime of appliances. | Tarpani and Gallego-Schmid: Providing digital health and well-being service to an average resident for 20 years; 718 kg, 741 kg and 1509 kg. |
| Mobile applications | Lokmic-Tomkins et al. 2023 | 2W: For example, services promoting health literacy, and warning about high temperature and air pollution incidents. Unclear if they decrease or add emissions, depends on the application and use cases. | NA |
| Diagnosis in hospitals | Fragão-Marques and Ozben 2023;  McAlister et al. 2020;  Schmidt and Bohnet-Joschko 2022 | D: For diagnostic tests, the use of new technologies can mitigate CO_2_e emissions (and reduce costs and  improve healthcare coverage). Appliances & software.  Point-of-care measurement (PoC) appliances instead of whole-blood sampling and measurements in laboratories. | McAlister:  49-116 g/ for other four tests,  but CRP 0.5 g/test |
| Overdiagnosis | Barratt and McGain 2021;  Barratt et al. 2022  Gray 2022; | D. Artificial intelligence could be used to detect and prevent overdiagnosis.  I: Large amount of data created by enhanced technologies can result in overdiagnosis, and this again to increased use of the technologies and IT infra. | Barratt et al 2022:  80% emissions from clinical care, 30% of clinical care is low-value care. In Australia, over 10 MtCO2e could be saved annually by eliminating harmful and low-value care. |
| Self-diagnosis using wearable sensors (see also telehealth above) | Lokmic-Tomkins et al. 2023;  Thompson 2021 | D: Heart rhythm monitoring instead of visit to health services. Also potential for early detection of diseases, to reduce the burden of healthcare.  2W: Increased number and energy use of the appliances and IT infra vs. diagnosis in health services. Large amount of digital data to store and analyse. | NA |
| Treatments in hospitals,  surgical  operations | Das and Chandra 2023;  Vafaei Sadr et al. 2024;  Zhang et al. 2022 | D: Low-carbon hospital infra. Optimizing space and patient treatment. Using AI to minimize use of facilities by eliminating unnecessary consultations (Das).  Use of deep learning in digital pathology. Means for reducing the carbon footprint of deep learning (related ICT processes). | Zhang et al:  32 kg per bed day;  263 kg per analysis. |
| Medical appliances (devices) | Lokmic-Tomkins et al. 2022;  Lokmic-Tomkins et al. 2023 | D: More efficiency by augmented intelligence, internet of things. Procure low-carbon appliances. More efficiency by robotic surgery.  I: Adding more machinery and its electronics, and more energy use. | NA |
| Robotics | Lokmic-Tomkins et al. 2022 | D: More efficiency by robotic surgery.  I: Add more machinery and its electronics, and more energy use. | NA |
| Internet of things (IOT) | Lokmic-Tomkins et al. 2023 | D: Efficiency of use of different appliances increased. Operations and processes of hospital infra optimized. | NA |
| Manufacturing medicines with printing | Elbadawi et al. 2023;  Li et al. 2023 | D: Optimizing energy use in pharmaceutical 3D printing through machine learning.  2W: The energy consumption values of 3D-printing medicines are comparable to conventional tablet production by powder compaction. The study highlights the roles of temperature, changing the design of tablets with CAD, and printer standby energy. | Elbadawi et al:  1-11 g per tablet  Reducing printing temperature:  decrease 6-33 % |
| Management of medicines storages and use | Fragão-Marques and Ozben 2023 | More efficient monitoring of inventories. Lower use of reagents. Personalized medicine. | NA |
| IT infrastructure and processes  in hospitals and other healthcare | Gray 2022;  Lokmic-Tomkins et al. 2022;  Lokmic-Tomkins et al. 2023;  Samuel and Lucassen 2022;  Thompson 2021 | D: Low-carbon IT-infrastructure. Energy- and resource-efficient server rooms and cloud computing, and renewable energy.  I: Added emissions by the increased volume of IT-infra and its use. | NA |
| IT services outside healthcare, cloud services | Das and Chandra 2023;  Lokmic-Tomkins et al. 2023 | Procurement of low-carbon cloud services | NA |
| Hospital building infrastructure (electricity use, heat use, water management) | Das and Chandra 2023;  Rahimi-Ardabili et al. 2022;  Schmidt and Bohnet-Joschko 2022;  Tennison et al. 2021 | D: Digital control systems for electricity and heat use. Energy consumption anomaly detection (Das).  D: Digital measurement and control of wastewater treatment.  D: For new buildings, design tools for optimizing space, function and carbon footprint. | NA |
| Electricity and heat: renewable energy | Gray 2022;  Kwon et al. 2024;  Lokmic-Tomkins et al. 2022;  Lokmic-Tomkins et al. 2023;  Samuel and Lucassen 2022 | D: Electricity is important component for the CF of nearly all the areas and functions. Procuring renewable low-carbon electricity, own production (solar, wind, geothermic heat). | NA |
| Transport of goods | Das and Chandra 2023;  Fragão-Marques and Ozben 2023 | D: More efficient logistics, digital control systems for low-carbon vehicles. | NA |
| Supply chains of production, manufacturing networks | Gray 2022;  Lokmic-Tomkins et al. 2022;  Samuel and Lucassen 2022;  Tennison et al. 2021;  Thompson 2021 | In articles, general discussion of the topic. | NA |
| Travelling of personnel, patients, visitors | Lim et al. 2013;  Mtioui et al. 2021;  Nicolet et al. 2022;  Palmer et al. 2023;  Tennison et al. 2021 | D: Digital services enabling shared taxis and carpools, decreasing the need for private cars and one-person taxis. Digital services enabling virtual care consultations and remote work of personnel.  Staff travel can be optimized through mathematical modelling. | 9 % of a national healthcare,  10 % of a hospital,  22 % of a hospital,  and 46 % of a private primary care |
| Leadership, planning and management of digitalization | Schmidt and Bohnet-Joschko 2022  Fragão-Marques and Ozben 2023  Lokmic-Tomkins et al. 2023 | Leadership in hospitals includes top-down strategic approaches as well as departmental and individual initiatives.  Digitalization must have adequate planning so that large scale implementation is possible without negative consequences.    D: Climate goals and low-carbon criteria in investments and procurement, innovative procurement, digitalization of operations (incl. work). | NA |

***The scales of the carbon footprint of digital healthcare solutions***

In the digitalization cases of our scoping review, the carbon footprint of in-person healthcare clinic visits ranged between 0.57 and 178 kgCO2e depending on the mode of transport used, while the footprint of virtual consultations ranged between 0.005 and 3 kgCO2e, the difference being 79–99 %. Similarly, an in-person continuous medical education meeting would cause emissions of 254 kgCO2e per participant, while a virtual event would cause emissions of 1 kgCO2e per participant. The carbon footprint of a diabetic eye exam was 0.00002–0.0002 kg, with autonomous artificial intelligence decreasing it by 80 %.

The carbon footprint values above can be put in the context of healthcare carbon footprints by benchmarking them with the review article on healthcare climate impacts written by Drew et al.^4^ The global average for national healthcare system emissions is around 300 kgCO2e per capita and year, ranging between 500 and 1000 kgCO2e in countries such as the UK and Finland, and it may reach up to 1800 kgCO2e in countries like the USA. The estimates for the climate impacts of surgical procedures have ranged between 5.9 and 1000 kgCO2e per single procedure. The estimates for the climate impacts of common radiological imaging methods have ranged between 1.1 and 20 kgCO2e per imaging study and for laboratory investigations between 0.0005 and 0.54 kgCO2e per test. The climate impacts of healthcare equipment have ranged between 0.0028 and 4.5 kgCO2e per use for reusable items and 0.0018 and 18 kgCO2e per use for single-use items. For medical interventions, the impact range was 0.018–28 kgCO2e per patient day. Another benchmark is also offered by the carbon footprint of a *bed-day*, found to have average carbon footprint of 125 kgCO2e in the UK^5^ and 32 kgCO2e in Germany^6^.

Other than telemedicine studies, patient and staff travel are not always included in carbon footprint assessments of healthcare services. Patient travel indeed is included in the carbon footprint of the household and including it in the carbon footprint of healthcare leads to double counting. Staff travel should be included in the carbon footprint of the healthcare. However, estimates have shown that patient and staff travel contribute to 10 % of the healthcare sector’s carbon footprint in England^5^, 9% of a hospital unit’s carbon footprint in Australia^7^, 22% of a hospital unit’s carbon footprint in Morocco^8^, and 46% of private primary healthcare practices’ carbon footprint in Switzerland^9^. Despite the variation, there is a sizeable contribution of patient and staff travel to the carbon footprint of healthcare services.

**References**

1 Lange O, Plath J, Dziggel TF, et al. A transparency checklist for carbon footprint calculations applied within a systematic review of virtual care interventions. *Int J Env Res Public Health* 2022; **19**: 7474. <https://doi.org/10.3390/ijerph19127474>

2 ISO (International Standard Association) 2024. ISO 14067:2018. Greenhouse gases — Carbon footprint of products — Requirements and guidelines for quantification. <https://www.iso.org/standard/71206.html> (accessed Jan 6, 2024)

3 IPCC (Intergovernmental Panel on Climate Change). (2018). Global Warming of 1.5° C. An IPCC Special Report on the impacts of global warming of 1.5° C above pre‐industrial levels and related global greenhouse gas emission pathways, in the context of strengthening the global response to the threat of climate change, sustainable development, and efforts to eradicate poverty. Geneva: IPCC. <https://www.ipcc.ch/sr15/>

4 Drew J, Christie SD, Rainham D, Rizan C. HealthcareLCA: an open-access living database of health-care environmental impact assessments. *Lancet* *Plan* *Health* 2022; **6**: e1000–12. <https://doi.org/10.1016/S2542-5196(22)00257-1>

5 Tennison I, Roschnik S, Ashby B, et al. Health care's response to climate change: a carbon footprint assessment of the NHS in England. *Lancet Plan Health* 2021; **5**: e84–92. <https://doi.org/10.1016/S2542-5196(20)30271-0>

6 Zhang X, Albrecht K, Herget‐Rosenthal S, Rogowski WH. Carbon footprinting for hospital care pathways based on routine diagnosis‐related group (DRG) accounting data in Germany: An application to acute decompensated heart failure. *J Industr Ecol* 2022; **26**: 1528–42. <https://doi.org/10.1111/jiec.13294>

7 Lim AE, Perkins A, Agar JW. The carbon footprint of an Australian satellite haemodialysis unit. *Aust Health Rev* 2013; **37**: 369–74. <https://doi.org/10.1071/AH13022>

8 Mtioui N, Zamd M, Ait Taleb A, Bouaalam A, Ramdani B. Carbon footprint of a hemodialysis unit in Morocco. *Ther Aphe Dial* 2021; **25**: 613–20. <https://doi.org/10.1111/1744-9987.13607>

9 Nicolet J, Mueller Y, Paruta P, Boucher J, Senn N. What is the carbon footprint of primary care practices? A retrospective life-cycle analysis in Switzerland. *Env Health* 2022; **21**: 1­­­­­­­­­­­­­­­­­­­–10. <https://doi.org/10.1186/s12940-021-00814-y>
